# Catalytic Reduction of Noble Metal Salts by Sodium Hypophosphite Promoted by the Film Poly-(*p*-Allyl Ether Benzenesulfonic Acid)

**DOI:** 10.5402/2011/759817

**Published:** 2011-04-11

**Authors:** M. I. C. F. Costa, J. R. Steter, F. L. S. Purgato, J. R. Romero

**Affiliations:** Departamento de Química, FFCLRP-USP, Avenida dos Bandeirantes 3400, 14040-901 Ribeirão Preto, SP, Brazil

## Abstract

Glassy carbon electrodes were coated with the film poly-(*p*-allyl ether benzenesulfonic acid) by an anodic procedure. Nickel, platinum, and palladium ions were introduced into the film by ion exchange of H^+^ with the corresponding salts. These ions were catalytically reduced to their corresponding metals using the known electroless reducing agent sodium hypophosphite. Scanning electron microcopy and energy dispersive X-ray spectroscopy were carried out to demonstrate the occurrence of the catalytic process. To compare this method with another one carried out in our laboratory, the electrocatalytic reduction of H^+^ was studied using the same modified electrodes. A suggested mechanism for the catalysis is proposed.

## 1. Introduction

Modified electrodes (MEs) coated with a polymeric film containing particles of noble metals dispersed in the film are useful for the accomplishment of electrocatalytic hydrogenation of organic substrates [1]. The polymeric film exchanges ions with the corresponding salts. These ions are then electrochemically reduced, and the obtained modified electrode (ME) containing noble metals is used as the working electrode for hydrogenation of organic substrates. By application of an adequate cathodic potential to the cell containing a mineral acid solution, the H^+^ ion is reduced to H^·^, which is adsorbed at metallic surfaces like Ni, Pd, and Pt. Organic compounds with double bonds as olefins, carbonylic functions, or aromatics are also adsorbed, and the transference of hydrogen to these species yields saturated compounds.

In our laboratories, we have studied the preparation of an electrode, modified with the film poly-(*p*-allyl ether benzenesulfonic acid) which is able to perform the electrocatalytic hydrogenation of organic substrates [1–3]. The film was prepared by electrochemical oxidation of the corresponding monomer, leading to initial formation of the radical cation, which in turn is the initiator of a chain reaction. The resulting polymer coats the electrode, giving rise to a film with high chemical and mechanical stability [4]. This film presents low conductivity but recovers its electroactivity when the sulfonic group changes H^+^ for Ni^2+^, Pd^2+^, or Pt^2+^. Some cathodic scans are enough to reduce these ions to metals in the fundamental state.

Mixed electrodes containing Cu, Fe, and Ni particles have been studied recently [5–10]. The electrodes were prepared by *electroless* or galvanic displacement of noble metals like Ni, Pd, and Pt, thereby yielding the modified electrodes ME Cu/Ni, Pd, or Pt, ME Fe/Ni, Pd, or Pt, and ME Ni/Ni, Pd, or Pt [3, 9, 10]. All of them perform in the electrocatalytic hydrogenation of organic substrates effectively. 

*The electroless *process consists of the adsorption of atomic hydrogen [11–19], provided by sodium hypophosphite, on the surface of Fe, Cu, Ni, Pd, or Pt particles previously present in the electrode. Then, an adequate potential must be initially applied to the electrode, dipped into the metal salt solution for some minutes, to produce the microcrystals necessary for the *electroless* process. The atomic hydrogens that are adsorbed on the electrode transfer electrons to the noble metal ions present in the solution, thereby reducing them to metallic particles. The latter particles become dispersed in the film, resulting in the growth of the previous crystals.

Interesting results from previous works have shown that such a film is able to catalyze the reduction of the metallic ions by sodium hypophosphite itself, in the absence of metal crystals. Ni^2+^, Pd^2+^, or Pt^2+^ ions are not reduced when their salts are dissolved in *electroless* solution; however, the reduction of these cations takes place fast and spontaneously when they are associated with the film poly-(*p*-allyl ether benzenesulfonic acid) with no need for H^·^ adsorption. 

This work describes the catalytic properties of the film poly-(*p*-allyl ether benzenesulfonic acid) in the reduction of noble metal ions to their corresponding metals by sodium hypophosphite.

## 2. Experimental

### 2.1. Reagents

All reagents and solvents used here were analytical grade and purified when necessary.

### 2.2. Equipments

A Potentiostat/galvanostat PAR model 273 and the software M270 program were employed in the experiments. The current was registered on an Intralab 2030 recorder. Cyclic voltammetry experiments were carried out in a cell (15 mL) using glassy carbon as the working electrode (3 mm diameter), an Ag/AgCl electrode as the reference electrode, and a platinum wire as the auxiliary electrode. Scans were carried out from 0.0 to −1.0 V versus Ag/AgCl, at 10 or 100 mV·s^−1^. 

### 2.3. Morphological Characterization

The surface morphology, microstructure, and elemental composition of the metals deposited on the film were analyzed by scanning electron microcopy (SEM) and energy dispersive X-ray spectroscopy (EDS), by means of a Leica-Zeiss LEO 440 model SEM coupled to an Oxford 7060 model analyzer.

### 2.4. Preparation and Characterization of the Modified Electrode

The preparation of the monomer *p*-allyl ether benzenesulfonic acid and its polymerization over a glassy carbon electrode (sulfonic acid film) has been described in the literature [4]. The modified electrode prepared by ion exchange/chemical reduction had been previously characterized by SEM and EDS.

### 2.5. Exchange of Metallic Ions with the Hydrogen of the Sulfonic Acid Film-Incorporation of Ni^2+^, Pd^2+^, and Pt^2+^ Ions into the Modified Electrode and Electrochemical Reduction of the Metallic Ions

A glassy carbon electrode (3 mm diameter) coated with the sulfonic acid film was dipped into 2.5 mL NiSO_4_ (0.12 mol L^−1^) solution for one hour. The electrode was washed with deionized water and installed versus Ag/AgCl in a cell containing 10 mL KCl (0.1 mol L^−1^) solution. Then, five scans from 0.1 to −0.7 V versus Ag/AgCl were carried out at 10 mVs^−1^. At the end of the procedure, the electrode was washed with deionized water.

The same procedure was also carried out in the cases of the platinum and palladium salts (0.12 mol L^−1^ PdCl_4_ or PtCl_4_ solutions).

These electrodes described above were used for the SEM-EDS characterization.

### 2.6. Hydrogen Generation from a Mineral Acid Solution

The modified electrodes containing dispersed noble metals were arranged in a cell containing 10 mL H_2_SO_4_ (0.1 mol L^−1^) solution. Scans from 0 to −1.0 V versus Ag/AgCl were recorded.

### 2.7. Polymeric Film Catalysis: Reduction of the Incorporated Metallic Ions by Sodium Hypophosphite and Hydrogen Generation from a Mineral Acid Solution

The carbon glassy electrode coated with the sulfonic acid film was dipped into 10 mL NiSO_4_ (50 mmol L^−1^) and NaH_2_PO_2_ (0.3 mol L^−1^) solution (pH adjusted at 2.0 with concentrated HCl) in different time periods. The electrode was gently washed with water and installed as the working electrode in a cell containing 10 mL H_2_SO_4_ (0.1 mol L^−1^) solution. Scans from 0 to −1.0 V were performed to generate hydrogen. In another set of experiments, the pH was adjusted to 8.0 with NH_4_OH. Both pH-adjusted solutions were dipped into the nickel salt solutions for different time periods, varying from 20 s to 25 min., and hydrogen generation was carried out in the same way.

The same procedure was employed in the case of sulfonic acid dipped into a solution of PtSO_4_ or PdSO_4_ (50 mmol·L^−1^), and 0.3 mmol L^−1^ NaH_2_PO_2_ during pre-established immersion periods, at pH 2.0 or 8.0.

## 3. Results and Discussion

The strategy employed here to demonstrate the catalytic effect of the sulfonic acid film containing dispersed Ni^2+^, Pd^2+^, and Pt^2+^ on the reduction of these ions using NaH_2_PO_2_ was to compare hydrogen generation by the modified electrodes containing Ni, Pd, and Pt particles prepared by two different methods: (A) the conventional approach consisting of ion exchange and electrochemical reduction of the corresponding ions and (B) dipping of the electrode containing the sulfonic acid film into an *electroless* solution containing the respective ions. Ion exchange occurs followed by chemical reduction of the metallic ion promoted by NaH_2_PO_2_ at pH 2.0 or 8.0. 

SEM and EDS analyses were carried out to show the presence of the metal particles in the film. 

**Figure 1:**
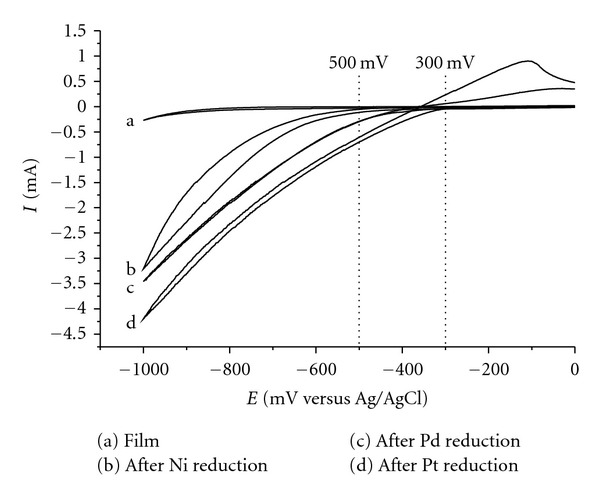
Cyclic voltammograms of hydrogen generation using (a) the film only, (b) Ni, (c) Pd, or (d) Pt prepared by ion exchange and electrochemical reduction (method A). Scan intervals from 0.0 to −1.0 V versus Ag/AgCl and scan rate 100 mVs^−1^.

Figure 1(a–d) depict the currents for hydrogen generation obtained by scans in H_2_SO_4_ solution, using the MEs with Ni^2+^ (b), Pd^2+^ (c), or Pt^2+^ (d) prepared by method A and designated ME Ni, ME Pd, and ME Pt.

Experimental conditions such as pH 7.0 and different immersion periods were applied, since these were the best results obtained in previous studies [2]. For both ME Pd and ME Pt, the onset of current due to H_2_ evolution took place at potential values, about 100 to 200 mV lower compared with ME Ni. The largest cathodic current was obtained for ME Pt (Table 1).

**Figure 2:**
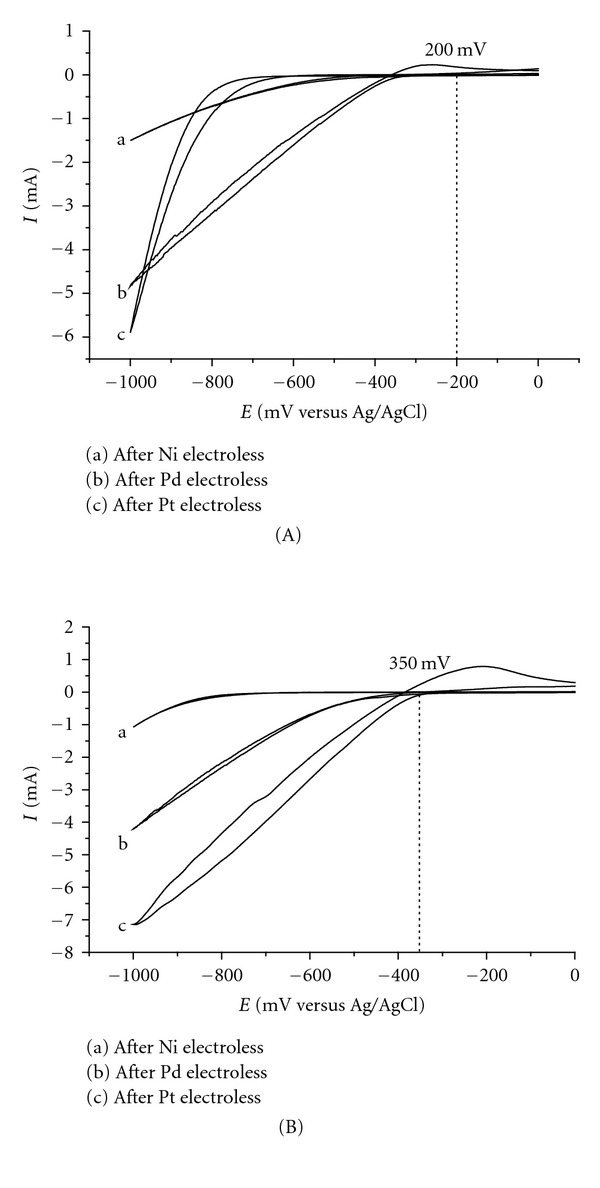
(A) Cyclic voltammograms of hydrogen generation by the MEs (a) Ni, (b) Pd, and (c) Pt prepared at pH 2 by ion exchange and chemical reduction (method B). Scan intervals from 0.0 to −1.0 V versus Ag/AgCl and scan rate 100 mVs^−1^. (B) Cyclic voltammograms of hydrogen generation by MEs (a) Ni, (b) Pd, and (c) Pt prepared at pH 8 by ion exchange and chemical reduction (method B). Scan intervals from 0.0 to −1.0 V versus Ag/AgCl and scan rate 100 mVs^−1^.

Figures 2(A) and 2(B) display the currents obtained with scans in H_2_SO_4_ solution, using the MEs Ni, Pd, and Pt prepared according to method B, at pH 2.0 and 8.0, using the time period necessary for current stabilization (maximum electrode efficiency). It is important to note that there is not anodic peak of reduction of metallic ions present in solution. The currents were similar and have the same order of magnitude than those generated by the MEs prepared by method A. The onset of H_2_ evolution in the case of the MEs Pd and Pt at pH 2 and pH 8 occurred at potentials 350 and 200 mV lower, compared with ME Ni. 

When method B and pH 2 are used to prepare the MEs, the highest currents are achieved with the MEs Pt and Pd (Table 1). When method B and pH 8 are employed, the highest current is obtained for ME Ni followed by ME Pt. These currents are higher than those achieved with the MEs prepared by method A. At both pHs, ME Pt stabilizes faster than the other MEs.

The onset of H_2_ evolution occurs at the same potential ME Ni, whatever the preparation method is (A or B). Hydrogen evolution started at lower potentials in the case of the MEs Pd and Pt prepared by method B, indicating that a lower energy is necessary. But this process was pH dependent, so one can conclude that this is due to the nature of the employed metals. Pd and Pt are generally considered more efficient than Ni. It is important to highlight that former studies [9, 10] have demonstrated that the electrochemical hydrogenation of organic substrates begins at potentials close to that for initial H_2_ generation. Substrate hydrogenation occurs with transfer of the radical hydrogen to the unsaturated organic molecule, which are both adsorbed on the surface of the noble metal. The excess of radical hydrogen at more negative potential produces bubbles that damage the integrity of the film, repel the approximation of the substrate during the electrolysis by convection, and are energetically unnecessary for the hydrogenation reaction. So, one can conclude that MEs prepared by method B are more efficient and simpler than those prepared by method A, considering the experimental preparation of the electrodes, the current of hydrogen generation, and the potential necessary for the onset of hydrogen radical production.

The different times to stabilize the electrodes in the hydrogen generation process are due to macromodifications of the charged polymer dipped in a solution with electrolyte.

### 3.1. EDS

**Figure 3:**
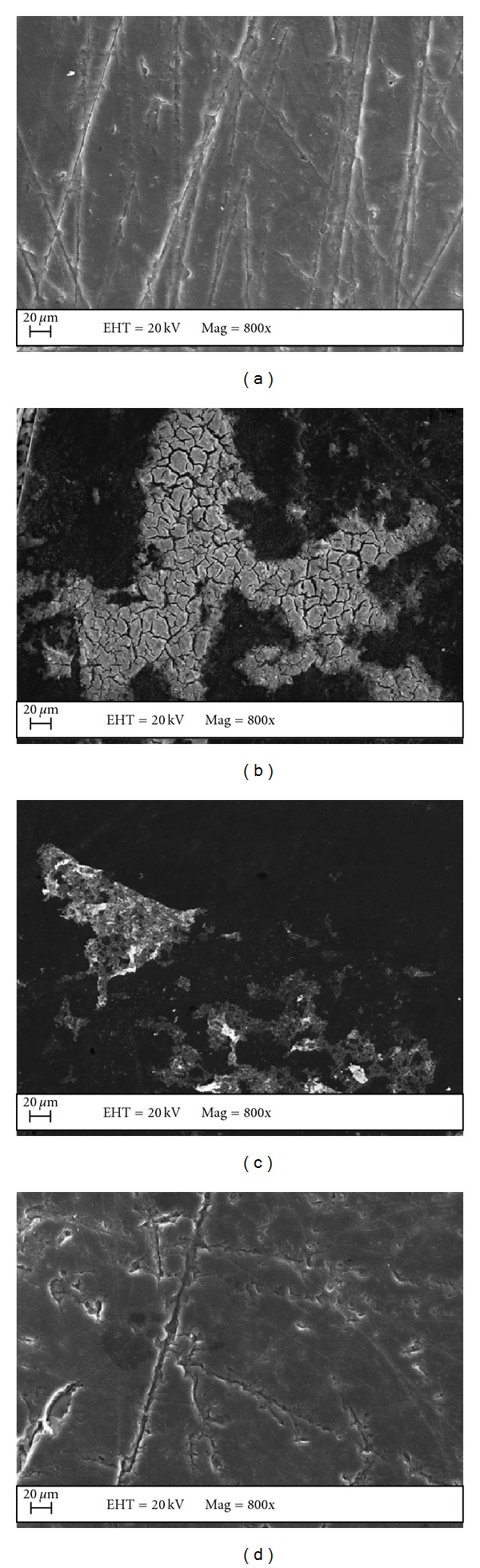
SEM image of the film and the metals deposited onto the glassy carbon surface; (a) ME film, (b) ME Pt, (c) ME Pd, and (d) ME Ni.

**Figure 4:**
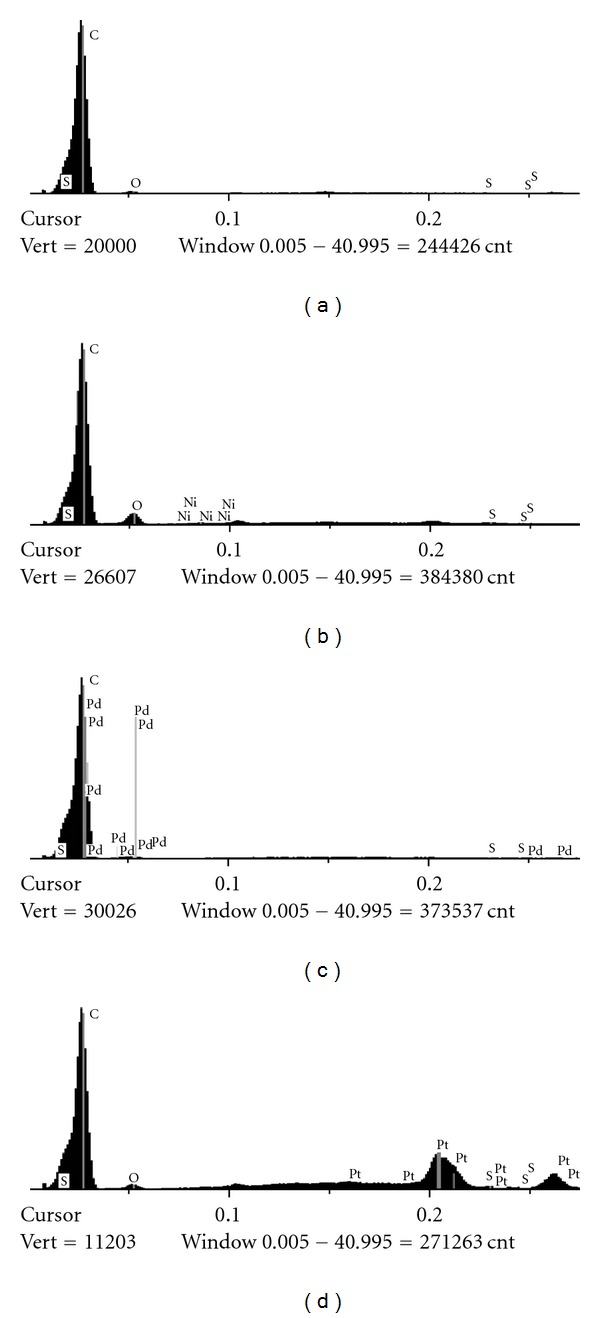
EDS spectra of (a) ME film, (b) ME Ni, (c) ME Pd, and (d) ME Pt. EDS composition of the electrodes is: ME Ni (film + metal) 0.12 mol L^−1^ Ni^2+^, Pt^2+^, and Pd^2+^.

The EDS analysis quantifies the percentage of metals in the MEs. Data for the elements carbon, oxygen, sulfur, and the metals Ni, Pd, and Pt are listed in Table 2 in the MEs Ni, Pd, and Pt prepared by method B. Figures 3 and 4 show the EDS results obtained for all the investigated electrodes prepared by method B. 

EDS gives anomalous results for carbon and oxygen, because the electrode contains carbon. Moreover, analysis reveals several peaks for metal oxides (Figure 4). For ME Pt, the occurrence of sulfur is smaller than the limit of detection ratio of the instrument. But it is interesting to consider the relation between the [M]/[S] ratio for the MEs Pd and Ni. In both cases, the occurrence of the metal is higher than that of sulfur. It is considered that there is one sulfur atom per monomer unit in the polymer. This fact evidences that the catalysis takes place in several cycles. This fact is evident, even for Pt^2+^, though not quantifiable. Again, one can remark here that Pt^2+^ is more abundant in the ME compared with Pd^2+^ and Ni^2+^, thereby explaining the high efficiency of ME Pt compared with MEs Pd and Ni.

### 3.2. Morphological Characterization

Figure 3 corresponds to the SEM image of the surface of the polymeric film (A), and polymeric film with metallic ions Pt, Pd, or Ni, (b, c, and d). EDS was carried out to confirm the presence of the metals (Figure 4).

Figure 3(a) shows the film roughness and evidences that fibers were formed after deposition of the film onto the glassy carbon (b, c, and d). It seems that the polymer covers all the surface of the electrode.  Figure 3(d) reveals the same effect, but one can notice the presence of cracks that increase with the amount of Ni metal incorporated into the film. Figures 3(b) and 3(c) demonstrate that the metals are agglomerated in the film with cracks and without cracks.

Figure 4(a) confirms the SEM results and the presence of carbon as the major constituent of the samples. The presence of oxygen and sulfur is negligible. Although the film is composed by C, O, S, and H, the major presence of C is due to the electrode support material, as can be seen in all the cases. The minor presence of Ni in Figure 4(b) is also due to the program system, which compares all the elements with C (the major element). The higher quantity of the metals Pd and Pt is in agreement with the results obtained previously, which showed that these electrodes are more reactive compared with ME Ni.

**Figure 5:**
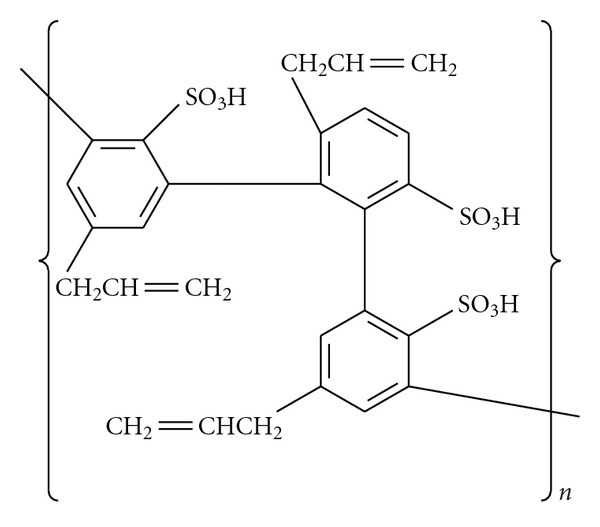
Partial structure of the polymer showing free sulfonate groups.

**Figure 6:**
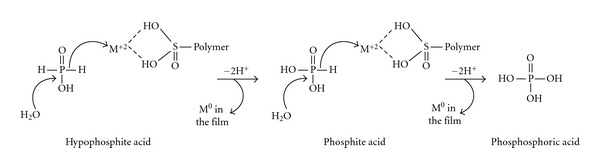
Mechanism proposed for the reduction of metal ions by hypophosphite acid in acid medium catalyzed by the polymer.

The mechanistic proposition for metal dispersion into the film is based on two major facts: (a) hypophosphite is a strong reducing agent, which able to supply hydride ions and then reduce the positive bivalent ions Ni^2+^, Pd^2+^, and Pt^2+^ to the corresponding M^0^; and (b) the capacity of the sulfonate groups in the film to fix these ions in their valence sphere. Here, it is necessary to indicate that in the film, the polymeric bonds involve the aromatic groups, leaving the sulfonate group free to associate with ions, as earlier demonstrated in the literature for tyramine electropolymerization [20] and illustrated here in Figure 5 as a simplified scheme. The products are phosphoric acid (or the corresponding sodium phosphate, depending on the pH) and M^0^, the metal crystals (Figure 6). Hypophosphite acid or sodium hypophosphite is attacked by two water molecules, being oxidized and furnishing two hydride ions that reduce the metallic ion. The binding of the metal ion by sulfonate or sulfonic groups is a well-known step in the catalytic process, since it also occurs in enzymatic catalysis. In acid or basic pH, there is probably a strong decrease in the solvation sphere of the ion for all the cations studied here, since the bulky polymer structure facilitates the approximation of the small hydride ion. This proposed mechanism is quite different from the “*electroless*” mechanism [14] based on the homolytic cleavage of the P–H bond, which gives radical hydrogen adsorbed onto the noble metal surface.

## 4. Conclusions

The film poly-(*p*-allyl ether benzenesulfonic acid) catalyzes the reduction of Ni^2+^, Pd^2+^, and Pt^2+^ to the corresponding M^0^ noble metals by binding the ions in the valence sphere of the free sulfonate group. Sodium hypophosphite transfers the electrons through the hydride ion when it is oxidized by water to phosphite or phosphoric acid. SEM and EDS analyses showed the presence of metal crystals dispersed in the film in an amount higher than the equivalent molecular polymer unit, which confirms the catalytic effect on the reduction reaction. The electrochemical hydrogen generation of two kinds of electrodes, one prepared by electrochemical reduction of the metal ions and the other by this catalytic process, showed that the latter method is more efficient than the former. The ME Pt is the most efficient in acid pH, whereas ME Ni is the most efficient in basic pH. When MEs are prepared by the catalytic process, less energy is necessary for initiation of the hydrogen generation useful for the catalytic hydrogenation of organic substrates, compared with the process involving the electrochemical preparation of MEs.

## Figures and Tables

**Table 1:** Currents (mA) of hydrogen generation and stabilization time using the MEs Ni, Pd, and Pt prepared at pHs 7.0, 2.0, or 8.0, using method A or B.

Currents for hydrogen generation (mA)					
Method A			Method B		
ion exchange/electrochemical reduction			ion exchange/chemical reduction-catalysis		
ME	pH 7	pH 2	time (min)	pH 8	time (min)
Ni	3.2	1.0	18	6.0	25
Pd	3.5	4.2	15	1.6	10
Pt	4.2	7.6	2	4.8	15

**Table 2:** Occurrence of the metals in the MEs, as determined by SEM-EDS (for method B).

ME	Elements (%)				[M] versus [S]
	C	O	S	Metal	
Pt	85.1	5.3	0.0^a^	9.5	—
Pd	96.7	2.6	0.01	0.5	[Pd]/[S] = 51.3
Ni	82.5	17.0	0.14	0.3	[Ni]/[S] = 2.2

^a^Lower concentration than the detection limit of the instrument (toward the maximum for carbon).
